# Pediatric Ovarian Torsion in an 8-year Old Girl; A Case Report Study

**DOI:** 10.31661/gmj.v13i.3060

**Published:** 2024-01-22

**Authors:** Samaneh Yazdanpanah, Malihe Mahmoudnia

**Affiliations:** ^1^ Supporting the Family and the Youth of Population Research Core, Department of Obstetrics and Gynecology, Faculty of Medicine, Mashhad University of Medical Sciences, Mashhad, Iran

**Keywords:** Pediatrics, Ovarian Torsion, Laparotomy, Conservative Surgery

## Abstract

Background: Ovarian torsion in children causes abdominal pain. The clinical symptoms resemble other abdominal diseases, such as appendicitis. It happens when the ovary twists on its ligamentous attachment, which stops blood from getting to the ovary. A fast diagnosis accompanied by a high level of clinical suspicion and immediate surgical intervention is obligatory to save the ovaries and avoid complications. Case Presentation: In this case study, we describe a case of ovarian torsion in an 8-year-old Iranian girl who came with persistent (non-colicky) right-side abdominal pain. After ultrasonographic examinations, she was identified with an enlarged heterogeneous right ovary indicative of ovarian torsion and underwent emergency laparotomy without oophorectomy. Conclusion: It may be challenging to determine the source of abdominal pain in pediatric patients, due to factors such as insufficiently comprehensive medical histories and examinations, limitations in con-ducting radiological procedures, as well as the comparatively diminished specificity of results in contrast to adult populations. If torsion is confirmed by ultrasonography, detorsion with\ without oophorectomy is advised for conservative treatment. This article presents a case study and a brief examination of the issues and complications associated with the identification and management of pediatric ovarian torsion.

## Background

The prevalence of ovarian torsion (OT) is found to be 4. 9 cases per 100,000 individuals among females aged 1 to 20 years, making it one of the rare gynecological emergencies in children and teenagers [1]. OT occurs when the ovary undergoes rotation on its ligamentous support, which may potentially obstruct blood circulation [2][3]. One belief is that OT occurs for roughly 3% of all cases of severe abdominal pain in pediatric patients [4]. While the prevalence of pediatric OT is reported to be as high as 52% among individuals aged 9-14, the incidence of neonatal OT for girls under the age of one is only 16% [5][6]. The OT mainly occurs in ovaries containing masses, including neoplasms and functional cysts. Clinical reports illustrated that right-side torsion is more prevalent [7][8]. This observed phenomenon could potentially be attributed to either a constricting sigmoid colon on the left side, which limits the range of motion, or a hypermobile cecum on the right side, which allows for increased flexibility [8].

OT is one of the medical emergencies requiring prompt diagnosis and immediate surgical intervention (e.g. detorsion) to prevent severe adnexal damage. Making a late surgical decision might cause ovarian dysfunction and ultimately increase the likelihood of complications, such as infection, necrosis, peritonitis, adnexal loss, or possibly death afterward [9][10]. The clinical manifestation of OT resembles other acute abdominal morbidities such as appendicitis. Hence, it is crucial to differentiate OT from other disorders. Diagnostic imaging modalities like the computed tomography (CT) scan, magnetic resonance imaging (MRI) scan, and Doppler ultrasound can be helpful in diagnosis, although they might also produce inconclusive results in OT instances [3]. Due to similarities in the signs and symptoms of OT to other abdominal diseases, its early diagnosis is sometimes tricky in young girls, especially for primary care physicians. This article aims to present a case of OT in an eight-year-old premenarchal girl with exclusive clinical symptoms, diagnosed on the eighth day of the illness and treated successfully with immediate surgical intervention. Since acute OT is a rare gynecological emergency in children while representing variable clinical symptoms among individuals, our case study can improve physicians’ expertise to efficiently differentiate OT from other abdominal pains through conducting imaging and physical examinations.

## Case Report

**Figure-1:**
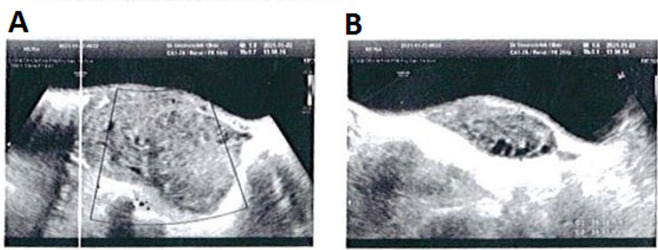
Figure 1. Ultrasound image of an 8-year-old girl with right ovarian torsion.

An eight-year-old girl, who was previously in good health, presented at the emergency department exhibiting acute right-sided abdominal pain that has persisted for one week. Pain was not alleviated by analgesics. She had no prior family history of ovarian illness, including OT.

She was examined twice by a pediatrician one week prior, during which postprandial vomiting was observed, without any concomitant symptoms of constipation, diarrhea, or urinary issues. She came to our hospital on the eighth day of the illness, after her abdominal pain had not been relieved by analgesics. She was rational and conscious, and not dehydrated but showed uncomfortably in all physical positions, including in supine, prone, and Fowler’s conditions, upon arrival to our department. She exhibited neither signs of recent nor severe neurological impairments.

During the physical examination, the patient exhibited vital signs within the expected range for her age. Specifically, the patient had a pulse rate of 85 beats per minute, a blood pressure reading of 100/60 mmHg, a respiratory rate of 20 breaths per minute, and an axillary temperature of 36. 2°C, and an oxygen saturation level of 99% when breathing regular room air. Additionally, there was no evidence of respiratory distress. On physical exam, the patient’s vital signs indicated normal parameters for her age; pulse rate of 85 beats/minute, blood pressure of 100/60 mmHg, respiratory rate of 20 breaths/minute, an axillary temperature of 36.2°C, and oxygen saturation of 99% on room air with no indication of respiratory distress. Her cardiovascular and pulmonary systems were thoroughly examined, but nothing untoward was found. Although the patient had slightly generalized abdominal tenderness, during the examinations, the patient did not exhibit any involuntary guarding, rebound tenderness, hepatosplenomegaly, or costovertebral angle tenderness. Furthermore, there were no classic signs of appendicitis, such as Rovsing’s and Psoas’s symptoms.

**Figure-2:**
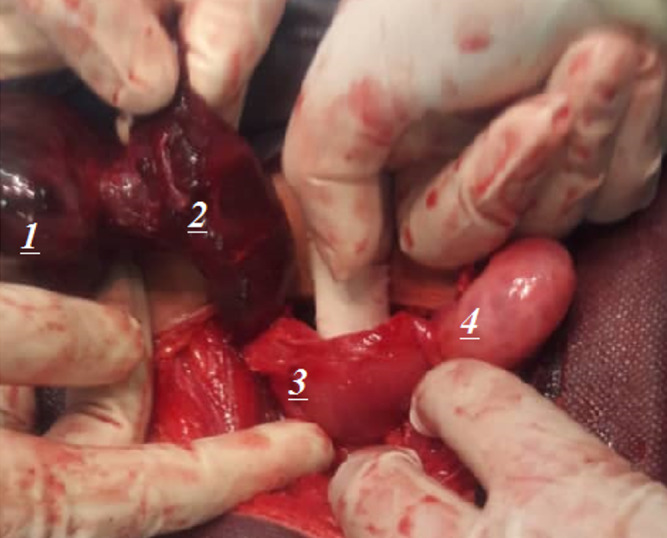
Figure 2. Right adnexal torsion observed during laparotomy on a child presenting with acute-severe and constant abdominal pain. The numbers show (1) right ovary (2) right fallopian tube (3) uterus and (4) left normal ovary.

Ultrasound imaging of the abdomen and pelvis indicated an enlarged heterogeneous right ovary measuring 7.1cm × 4.2cm × 3cm, with no indication of vascular flow on color Doppler evaluations, reflecting an ovarian torsion diagnosis (Figure-1). Furthermore, the uterus was not visible in grayscale ultrasonic images due to the right ovary’s displacement from its typical location. The cul-de-sac exhibited a complete absence of free pelvic fluid. During an emergency laparotomy, the adnexa on the right side presented with torsion and discoloration, while the ovary on the left side exhibited normal characteristics (Figure-2). We detorted the adnexa but did not perform oophoropexy for it. Upon examining the right ovary’s perfusion, it was discovered to be intact with the fresh vascular flow but just a minor shift in the color of the adnexal tissue to pink.

The surgery was successfully finished without any specific complications. The pain at the incision sites was the only postoperative complaint\symptom that was well controlled with oral analgesics. She began receiving intravenous cefazolin 50mg/kg/day and metronidazole 7.5mg/kg/dose every six and eight hours respectively, for two days. The patient was allowed home after a successful recovery on the second postoperative day. The six-month follow-up examination did not identify any distinct complications in relation to the right ovary. However, the ultrasound imaging does not demonstrate a normal vascular flow and follicular pattern for the right ovary despite heterogeneous echogenicity, measuring 1.9 cm × 1.7 cm × 3.2 cm in diameter. We obtained the consent form from the patient and the case report ethical approval from the Mashhad University of Medical Sciences Ethics Committee, specifically with reference to IR. MUMSREC1401287.

## Discussion

Acute OT is an uncommon but critical issue in children. It may present in a normal ovary devoid of a primary mass. It is more likely to happen if the person displays traits such as having an ovarian mass or lesion, an enlarged ovary, being of reproductive age, being pregnant, undergoing ovulation induction, having a history of ovarian torsion (OT), undergoing tubal ligation, or being diagnosed with polycystic ovarian syndrome [11]. Pediatric OT is a medical emergency that needs to be diagnosed and treated promptly. According to studies, the mean age of pediatrics suffering from OT ranges between 9 to 12.5 years [2][6][12][13][14][15][16][17]. OT is diagnosed clinically but confirmed surgically. Due to its rarity and non-specific clinical and investigative results, it might be difficult to diagnose. Its symptoms vary from person to person and may include nausea, vomiting, diarrhea, and fever, in addition to the most frequent complaint, abdominal pain [18][19][20]. In pediatric patients presenting with OT (or "oppositional defiant disorder"), the manifestation of abdominal pain can display various characteristics. This discomfort may exhibit as either continuous or sporadic, with or without radiation to other areas of the body. Additionally, it can range in intensity from moderate to severe and persist for a duration spanning several hours to several days [3]. In our case, the patient presented suddenly with constant hypogastric pain, preferably on the right side of her lower abdomen, less than 48 hours before examinations. Other symptoms of OT such as vomiting and nausea can present similarly to various other medical conditions, including appendicitis, urinary tract infections, renal colic, gastroenteritis, and other diseases causing abdominal and pelvic pain [21].

The clinical presentation exhibits variability among individuals presenting with an acute surgical abdomen, the presence of a palpable tumor, and an increased leukocyte count [18][19][20]. It can be said that history, clinical symptoms, and imaging can play a crucial role in diagnosing OT. Ultrasound is a standard imaging technique to diagnose OT since it can evaluate ovarian architecture and perfusion rapidly and non-invasively [22]. Ultrasound detects adnexal torsion 92% sensitively and 96% specifically [23]. Ultrasonography has been identified as an effective method for detecting torsion, wherein the absence of Doppler artery flow and ovarian enlargement serves as typical characteristics [5][24]. However, it is important to note that these features may not be consistently present [5][24]. The preservation of Doppler arterial flow in cases of acute torsion is predominantly observed due to the initial alteration of venous and lymphatic flow. The exclusion of torsion in ovarian torsion (OT) cannot be definitively determined solely through the utilization of normal Doppler results [5][25]. Ultrasound findings indicative of OT include enlarged ovaries, peripheral follicle distribution, aberrant adnexal position, and free fluid [23][25][26]. Furthermore, a coiled or twisted vascular pedicle detected on ultrasonography (known as whirlpool signs) indicates torsion [27][28]. Although ultrasound might be helpful, a clinical evaluation is still necessary to diagnose OT. In this particular case, the ultrasound examination revealed an abnormality characterized by an increased size of the right ovary and diminished blood flow as detected by Doppler imaging. This finding, in the context of the patient's presenting symptom of abdominal pain, prompted a diagnosis of right ovarian torsion prior to surgical intervention.

Adjuvant imaging modalities, including magnetic resonance imaging (MRI) and computed tomography (CT), can clarify ovarian anatomy and rule out alternative diagnoses [29]. For example, CT may identify OT if done before ultrasonography [11]. It detects the ovarian size, pedicle twisting, dilation of the pedicle, presence of lesions, edema, accumulation of free fluid as well as signs of hemorrhagic infarction and necrotic tissue. Despite their advantages, CT and MRI aren't practical in resource-poor settings [30].

Traditionally, ovarian torsion was treated by resecting the whole ovary without distortion [2]. Clinicians were of the belief that a hemorrhagic ovary comprised nonviable tissue and that conventional detortion alone would lead to thrombosis. Another concern was leaving a malignancy in the operation place [14]. However, there is no empirical data indicating that ovarian detorsion leads to an increased risk of complications (such as thrombosis, infection, reoperation, or undetected cancer) [1][31][32]; hence this potential risk should not impact surgical decision-making [31].

Despite gross appearance after surgery [13][33][34], follow-up examination has shown healthy and well-perfused ovaries up to 72 hours after distortion [35]. The ovary was also successfully saved with simple adnexal distortion in our patient. OT surgery may involve oophoropexy. In this method, the ovary is affixed to either the peritoneum, the uterosacral ligaments, or the round ligaments. Oophoropexy's advantages are debatable. Some think ovarian stabilization might damage the ovary and the follicle-oviduct connection [26][36][37]. Furthermore, torsion may make oophoropexy more difficult due to ovarian edema and size [38]. Experienced surgeons advocate for implementing oophoropexy in instances of recurring torsion, absence of the opposite ovary, elongated ovarian ligament, and torsion of the normal adnexa [26][36][39]. We didn't conduct an oophorectomy for the patient's right ovary as it was the first incidence of torsion.

## Conclusion

OT is an uncommon cause of stomach aches and may cause ovarian and fallopian tube infarction. Timely imaging and surgery are needed to avoid ovarian torsion complications. Ultrasound is the best imaging modality for diagnosing OT. Urgent adnexal detorsion with\without oophoropexy is recommended. Our data support the adoption of this conservative method which salvages the ovary with the lowest subsequent complications. The instance provided here explains one example that may contribute to more understanding of OT in children.

## Conflict of Interest

All authors declare no conflict of Interest.
